# Gut Microbiota in Lactose Intolerance: A Mendelian Randomization Study on Microbial Mechanisms and Potential Links to Tumor Inflammatory Microenvironments

**DOI:** 10.1155/mi/8181816

**Published:** 2025-06-05

**Authors:** Ya Xie, Qiongjiao Cao, Zhen Huang, Xin Zou

**Affiliations:** ^1^Department of Gastroenterology, The First People's Hospital of Loudi, Loudi, China; ^2^Intensive Care Unit, The First People's Hospital of Loudi, Loudi, China; ^3^Suizhou Hospital of Traditional Chinese Medicine, Suizhou, China; ^4^Department of Gastroenterology, Ganzhou Hospital of Guangdong Provincial People's Hospital, Ganzhou Municipal Hospital, Ganzhou, China

**Keywords:** gut microbiota, inflammation, lactose intolerance, mendelian randomization, tumor microenvironment

## Abstract

**Background:** Previous observational studies have suggested an association between the composition of the intestinal microbiome and lactose intolerance (LI). However, the causal direction remains unclear. This study utilized Mendelian randomization (MR) to rigorously evaluate the potential causal link between the gut microbiome and LI.

**Methods:** Genome-wide association studies (GWASs) summary statistics for gut microbiota and LI were sourced from previously published GWAS studies. Multiple methods, such as Simple mode, MR-Egger regression, weighted median, inverse variance-weighted (IVW), and weighted model, were used to determine the causal relationship between gut microbiota and LI. To validate the primary findings from the MR analyses, several sensitivity analyses were conducted. Furthermore, a reverse MR analysis was executed on bacterial taxa previously identified to have a potential causal link with LI risk, aiming to evaluate the possibility of reverse causation.

**Results:** The IVW results revealed that the genus *Lachnospiraceae UCG008* (OR = 0.584, 95%CI 0.356–0.958, *p*=0.0330), genus *Eubacterium hallii* group (OR = 0.467, 95% CI 0.242–0.899, *p*=0.023), and genus *Ruminococcus gauvreauii* group (OR = 0.506, 95% CI 0.2653–0.968, *p*=0.039) have a protective effect against LI. In contrast, the genus *Holdemania* (OR = 1.86, 95% CI 1.105–3.131, *p*=0.0194) displayed a predisposing effect. Sensitivity analyses did not detect any outlier single-nucleotide polymorphisms (SNPs). Further analyses reinforced the association between specific gut microbiota compositions and LI. No evidence suggested reverse causality between LI and the bacterial taxa identified in the reverse MR analysis.

**Conclusions:** From a genetic standpoint, this MR study indicates a causal relationship between variations in gut microbiota composition and LI. This not only underscores the potential of gut microbiota-centric treatments for LI but also provides a foundation for exploring the role of gut microbiota in LI development. Further study of the mechanism of Lachnospiraceae in the treatment of IL is conducive to the discovery of new therapeutic targets for IL.

## 1. Introduction

Persons with Lactose intolerance (LI) are unable to digest significant amounts of lactose [1]. LI manifests as gastrointestinal symptoms, such as stomach pain, bloating, and diarrhea, particularly in individuals with lactase nonpersistence (LNP) following lactose ingestion [2]. These symptoms likely arise from the inability to absorb lactose due to reduced lactase enzyme levels in the small intestine [3]. The prevalence of LI is influenced by the amount of lactose consumed, the expression of the lactase enzyme, and the composition of the intestinal microbiota [4]. LI is a widespread condition, predominantly caused by decreased β-galactosidase enzyme activity, which leads to the accumulation of undigested lactose in the digestive system [5]. The global prevalence of LI ranges from 57% to 65% [6], affecting ~70% of the world's population, especially in Latin American, Asian, and African countries [4]. Consuming dairy often results in persistent discomfort for these individuals, leading to the avoidance of dairy products. This can result in a decreased intake of essential nutrients, potentially impacting overall health [7].

In recent years, probiotic formulations and prebiotic products have emerged as promising therapeutic strategies for lactase deficiency due to their ability to modulate gut microbiota, enhancing lactose digestion and colonic compensation [8, 9]. Indeed, consuming yogurt containing active bacteria has been shown to enhance digestion and mitigate symptoms in lactase-deficient individuals [10]. Additionally, unprocessed yogurt intake correlates with improved lactose absorption [10]. However, undigested lactose might act as a beneficial prebiotic, fostering a healthier gut microbiome [11]. It's worth noting that incorporating probiotics and prebiotics doesn't replace the need for lactose-free products.

In managing their condition, individuals with LI may opt for lactase enzyme supplements [5]. However, the efficacy of these supplements remains debated [6]. Emerging evidence suggests a significant relationship between the intestinal microbiome and LI [11]. Probiotics found in both fermented and nonfermented dairy can alleviate the clinical symptoms of LI [4]. Specific probiotic strains have been proposed to assist in lactose digestion due to their inherent β-galactosidase activity [6]. A prospective placebo-controlled study revealed that after an 8-week treatment with probiotics or a placebo, both symptom questionnaires and lactose hydrogen breath test (LHBT) scores exhibited baseline deviations. Notably, the probiotic group reported a more pronounced symptom reduction post-lactose challenge compared to the placebo group [5].

Recent studies have shown that the microbial imbalance may exacerbate the pathological process through the release of proinflammatory factors and damage to the intestinal barrier [12]. It is worth noting that the interaction between the gut microbiota and the host immune system may affect disease progression by regulating cell death pathways, but the underlying molecular mechanisms remain unclear [13]. In addition, chronic intestinal inflammation is closely related to the reprograming of the tumor microenvironment, and microbiota-mediated immune-metabolic signaling may become a potential bridge connecting LI and diseases, such as colorectal cancer [14, 15].

Mendelian randomization (MR) is a technique that leverages data from genome-wide association studies (GWASs) to explore the causal relationships between risk factors and outcomes [16]. In MR design, genetic variations determined at conception are employed as instrumental variables to mitigate confounding and bridge evidence gaps [17]. Three pivotal assumptions underpin MR methods. First, instrumental variables (IVs) must be associated with the relevant risk factors. Next, these variables shouldn't be affected by confounders that influence both risk factors and outcomes. Lastly, outcomes should only be impacted by risk factors in the presence of IVs [18, 19].

Multiple studies over the years suggest a potential association between the gut microbiome and LI. However, biases and confounders in conventional observational research challenge the establishment of a direct link between specific bacterial taxa and LI risk [20]. A number of MR studies have found that gut microbiota is causally associated with dementia, eclampsia, and autoimmune diseases [21–23]. To elucidate the causal effect of the intestinal microbiome on LI susceptibility, we conducted a novel bidirectional two-sample MR study. To minimize potential microbiota influence on LI, we selected genetic variations strongly associated with distinct gut microbe populations as IVs.

## 2. Methods

### 2.1. Data Sources

**Figure 1:**
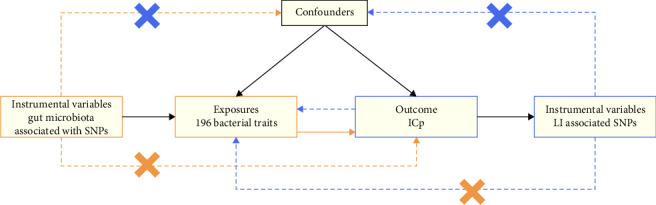
Study design of the bidirectional MR study of the associations between gut microbiota and LI.

Data on the gut microbiota composition in humans was sourced from a meta-analysis of GWASs by the MiBioGen Consortium [18, 20]. This aimed to investigate the influence of human genetics on gut microbiota. The study analyzed the 16S rRNA gene sequencing profiles and genome-wide genotypes of 18,340 individuals from 24 cohorts, primarily of European descent [24]. During this research, we excluded 15 bacterial taxonomic units that were unclassified or poorly annotated. Therefore, the study included 196 bacterial taxa (119 genera, 32 families, 20 orders, 16 classes, and 9 phyla) for analysis. The GWAS summary data for LI, including 445 cases and 198,259 controls of European ancestry, were provided by the FinnGen collaboration. A summary of the bidirectional MR design is shown in Figure 1.

### 2.2. Choosing IVs

IVs were selected based on specific criteria: IVs demonstrating a strong association with particular bacterial classifications (*p* < 1.0 × 10^−5^) were chosen as IVs [25]. To refine single-nucleotide polymorphisms (SNPs) associated with each bacterial group, only independent SNPs were retained. This refinement process employed a clumping distance of 10,000 kb and a linkage disequilibrium (LD) threshold for clumping set at *r*^2^ < 0.001, following standard practices to minimize LD bias. LD calculations were conducted using European samples from the 1000 Genomes Project as a basis. In cases where the desired variants were absent in the summary statistics, when exposure-related SNPs were missing from the outcome GWAS, we selected proxy SNPs exhibiting strong correlation with the target variant (*r*^2^ > 0.8) by searching the SNiPA database to facilitate the MR analysis [26]. Notably, palindromic SNPs and those with incompatible alleles were excluded from the MR analysis. The effectiveness of these IVs was assessed using the F-statistic, with an F-statistic significantly exceeding 10 indicating minimal bias risk due to weak IVs [27].

### 2.3. Statistical Analysis

Inverse variance-weighted (IVW) is the most widely used MR analysis method, which has the best statistical power, but it is assumed that all mutations are effective tools [28]. If there is directional pleiotropy, the estimated value is biased [29]. MR-Egger quantifies directional pleiotropy and explains it [30]. Even if all SNPs have pleiotropic effects, they can provide unbiased estimates. However, this method is sensitive to outliers and has low efficiency [30]. Weighted median is robust to outliers; when up to half of the SNP violates the instrumental variable hypothesis, it can provide unbiased estimates, but the efficiency may be low [28]. Therefore, we mainly use the IVW method for analysis, weighted median, and MR-Egger methods as a supplement. If there was heterogeneity among SNPs, a random-effects model was applied [31]. Cochran's Q statistics assessed IV variability. To comprehensively evaluate risk factor influence on results, alternative methods like simple mode [32], MR-Egger regression [30], weighted median [28], and weighted model [28] were used, even though they have lesser statistical power than the IVW method.

Sensitivity analyses were performed to evaluate the causal link between gut microbiota and LI. These included the MR-Egger regression, MR-Pleiotropy Residual Sum and Outlier method (MR-PRESSO) analysis, and leave-one-out sensitivity analysis. The MR-Egger's intercept provides a formal test for directional pleiotropy [30]. MR-PRESSO can detect abnormal SNPs that may exist in the data, which can make the analysis results more robust and reduce the interference of abnormal data on the overall conclusion. The MR-PRESSO analysis identifies and rectifies pleiotropic biases due to horizontal pleiotropy by removing outliers [33]. In the leave-one-out sensitivity analysis, each SNP was removed sequentially to detect any that significantly influenced outcomes. Leave-one-out can evaluate the impact of a single instrumental variable and test the reliability of the results. Additionally, reverse MR analyses ascertained potential reciprocal causal relationships between bacteria identified in the forward MR analysis and LI.

All statistical analyses were conducted using R (version 4.2.3), incorporating the TwosampleMR [34, 35] and MR-PRESSO packages [33].

## 3. Results

### 3.1. The Impact of Gut Microbiota on LI

**Figure 2:**
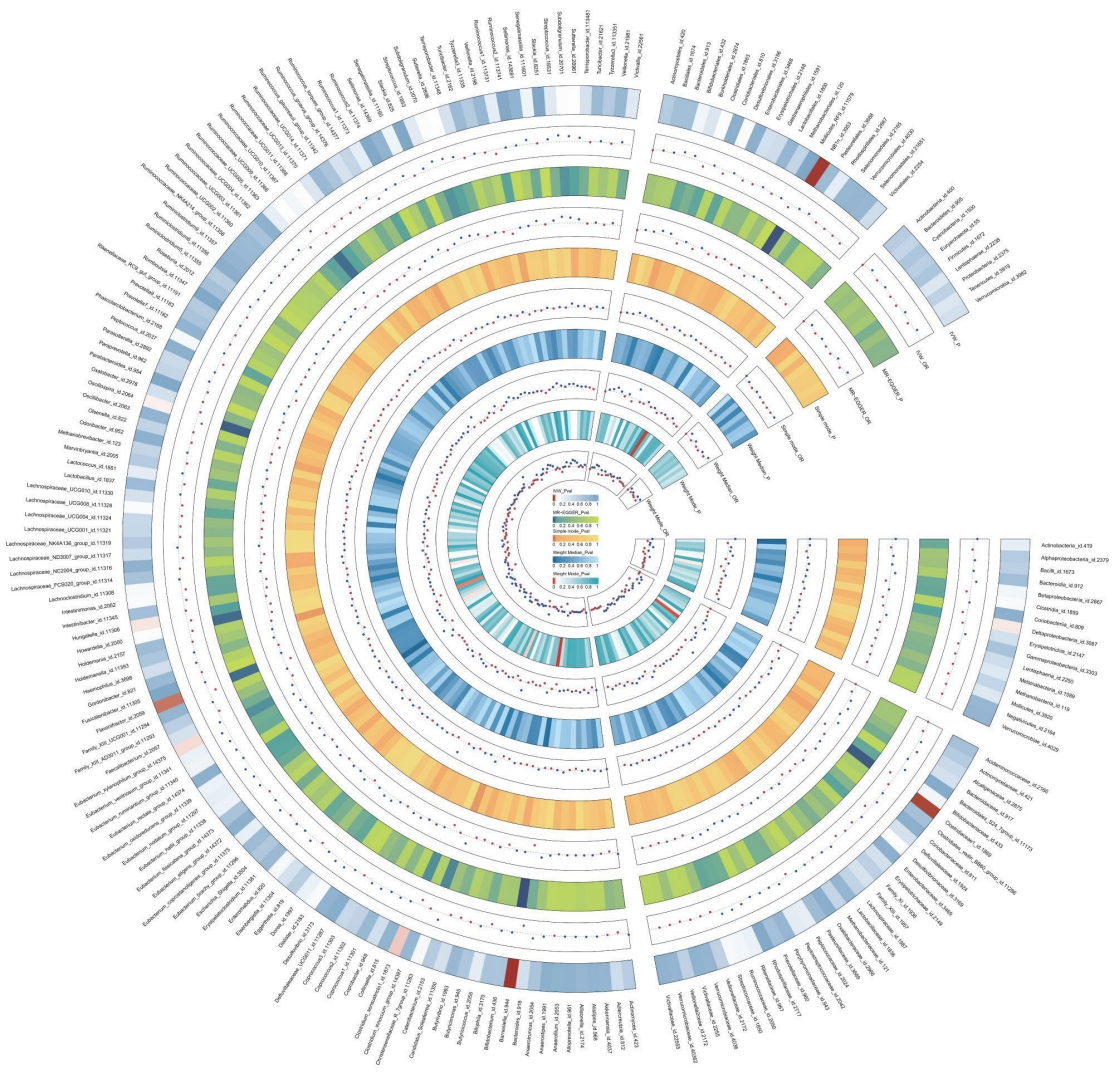
The circus plot showing five method results of all gut microbiota.

A total of 196 intestinal flora were examined using 2104 SNPs as IVs according to the IV selection criteria. The analysis results of MR on IVs are shown in a circle plot (Figure 2). In addition, Details about the specific genetic variations utilized for the MR analysis are provided in Supporting Information 2: Table S1. All the selected SNPs presented F-statistics exceeding 10, ensuring that the study was robust against the influence of weak instrumental variable bias.

**Figure 3:**
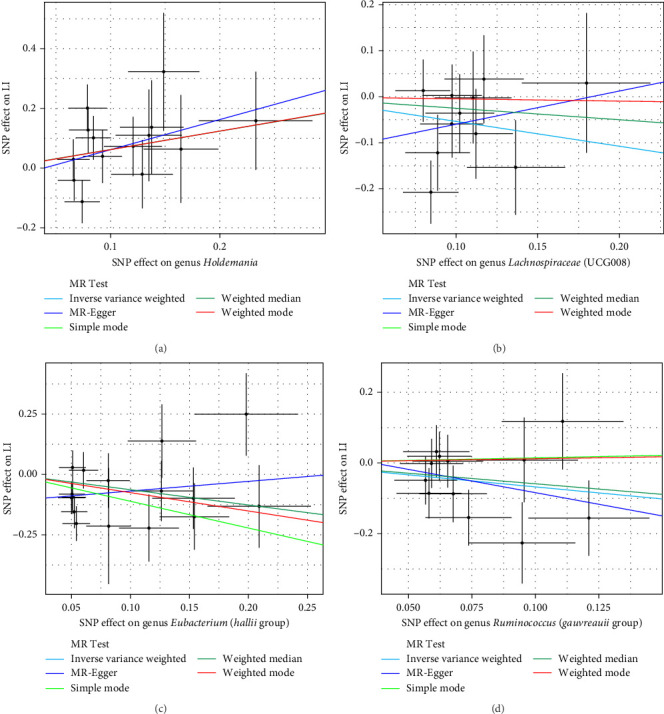
Scatter plots for the causal effect of gut microbiota on LI. (A) genus *Holdemania*,(B) genus *Lachnospiraceae* (UCG008), (C) genus *Eubacterium* (*hallii* group), and (D) genus *Ruminococcus* (*gauvreauii* group). Plot showing the effect sizes of the SNP effects on LI (*y*-axes) and the SNP effects on bacterial traits (*x*-axes).

As shown in Table 1 and Figure 3, there are four gut microbiota classifications, and at least one MR method identified associations between the risk of LI and two to four bacterial types. These include the genus *Holdemania*, genus *Lachnospiraceae* UCG008, genus *Eubacterium hallii* group, and genus *Ruminococcus gauvreauii* group. Specifically, IVW analysis results demonstrated a positive correlation between the genus *Holdemania* and the risk of LI (OR = 1.86, 95% CI:1.105–3.131, *p*=0.0194). Conversely, the risk of LI was inversely correlated with the relative abundance of the genus *Lachnospiraceae* UCG008 (OR = 0.584, 95%CI: 0.356–0.958, *p*=0.0330), genus *E. hallii* group (OR = 0.467, 95% CI: 0.242–0.899, *p*=0.023), and the genus *Ruminococcus gauvreauii* group (OR = 0.506, 95% CI: 0.2653–0.968, *p*=0.039).

### 3.2. Sensitivity Analysis

**Figure 4:**
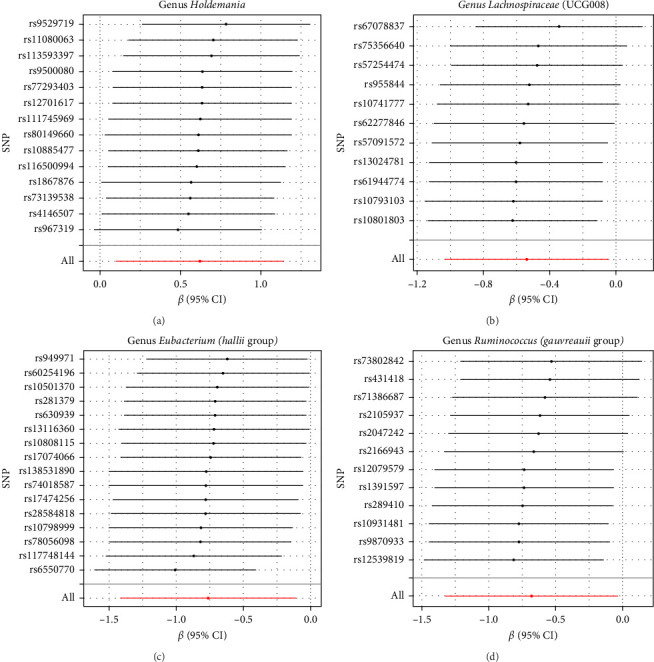
Leave-one-out plots for the causal effect of gut microbiota on LI. (A) genus *Holdemania*, (B) genus *Lachnospiraceae* (UCG008), (C) genus *Eubacterium* (*hallii* group), and (D) genus *Ruminococcus* (*gauvreauii* group).

The Cochran IVW Q test indicated minimal heterogeneity in the selected IVs (Supporting Information 2: Table S2). The MR-Egger intercept evaluation revealed no signs of horizontal pleiotropy (Supporting Information 2: Table S3). In the assessment of genus *Lachnospiraceae* UCG008 (*p*=0.440), Eubacterium genus *E. hallii* group (*p*=0.080), and genus *Ruminococcus gauvreauii* group (*p*=0.560), no pleiotropic SNP was detected having a protective impact against LI. However, the *Holdemania* genus was evaluated with a *p*-value of 0.470 through MRPRESSO analysis. The results from MR-Egger's intercept showed no significant findings, confirming the absence of horizontal pleiotropy (Supporting Information 2: Table S3). During the leave-out sensitivity evaluation, it was determined that the association between specific bacterial taxa and LI risk wasn't driven by an individual SNP (Figure 4).

### 3.3. Reverse MR Analysis

To ascertain the possibility of a causal relationship between LI and the relative abundance of four bacterial taxa, a reverse MR analysis was conducted. Significant SNPs (*p*  < 5 × 10^−8^) associated with the risk of LI were selected as IVs, as detailed in Supporting Information 2: Table S4. The analysis in Supporting Information 2: Table S5 revealed no bi-directional causal relationship between LI and the identified bacterial traits. The IVW test's statistical analysis confirmed the consistency among the chosen IVs, further detailed in Supporting Information 2: Tables S6 and S7. Our findings suggest that the link between LI and specific gut microbiota taxa is not driven by a single SNP, as visualized in Supporting Information 1: Figure S1.

## 4. Discussion

As per our current comprehension, this research represents a novel approach in using available GWAS summary data for a dual-direction MR analysis, delving into the causal ties between gut microbiota and LI risk. Our observations underscore an enhanced LI risk linked with a pronounced presence of the genus *Holdemania*. In contrast, augmented levels of genus *Lachnospiraceae* UCG008, genus Eubacterium *hallii* group, and genus *Ruminococcus gauvreauii* group suggested a diminished predisposition to LI.

The Lachnospiraceae, an anaerobic bacterial family within the class Clostridia, has emerged as a pivotal player in gut homeostasis [36]. Mounting evidence firmly establishes gut-resident Lachnospiraceae as the principal producers of butyrate, a short-chain fatty acid (SCFA) renowned for its ability to fortify the epithelial barrier integrity [37, 38]. Specifically, the g_Lachnospiraceae_NK4A136_group has been identified in multiple investigations to confer salutary effects on intestinal barrier function, thereby contributing to the maintenance of gut health [39, 40]. Members of the Lachnospiraceae family exhibit remarkable metabolic versatility, particularly in the fermentation of complex polysaccharides. These include glucans, mannans, xylans, galactans, pectins, and arabinans, which are depolymerized through the action of a diverse repertoire of carbohydrate–active enzymes (CAZymes) [36, 41]. Each CAZyme is specifically tailored to cleave distinct glycosidic linkages, enabling the efficient degradation of complex glycans [36]. Leveraging these metabolic capabilities, live biotherapeutic products (LBPs) incorporating Lachnospiraceae are currently under development as potential therapeutic modalities for metabolic syndrome and inflammatory bowel disease [36]. Notably, the present study has identified members of the Lachnospiraceae family within three beneficial bacterial consortia, underscoring their potential significance in modulating gut microbiota–host interactions.

In the context of IL, the equilibrium between the rates of removal and production of osmotic-active substances, including lactose and its intermediate metabolites such as lactate, within the colon is considered a critical determinant of symptom manifestation [42]. *E. hallii*, a well-characterized lactate-utilizing bacterium, plays a pivotal role in this metabolic interplay [43]. This species is capable of metabolizing acetate and lactate to produce butyrate and hydrogen, contributing to the generation of two essential SCFAs, propionate and butyrate [44]. These SCFAs exert multifaceted beneficial effects on gut physiology, including stimulating mucus production, promoting enterocyte proliferation and differentiation, and supporting epithelial cell homeostasis [45]. The putative protective role of *E. hallii* against IL is hypothesized to be associated with its capacity to metabolize lactose intermediate metabolites and maintain intestinal mucosal integrity. Ruminococcus gauvreauii, a member of the Lachnospiraceae family, exhibits unique metabolic characteristics. It can utilize various sugar alcohols, such as D–sorbitol, D–mannitol, and inositol, as carbon sources, with acetate being the primary fermentation end-product [46]. Lachnospiraceae UCG008 remains in its nascent stage. Although emerging evidence suggests associations with obesity and periodontal diseases [47, 48]. The mechanistic underpinnings of its potential causal relationship with IL remain largely elusive, warranting further in-depth investigation.

Genus *Holdemania* has the ability to metabolize sugars and can ferment glucose to produce lactic acid [6]. Studies have found that it may be correlated with clinical indicators of an impaired lipid and glucose metabolism [49, 50]. And the relative abundance of Holdemania increased significantly in irritable bowel syndrome and other diseases [51]. However, there is still a lack of relevant research in LI.

The mechanisms and potential clinical applications for targeting these flora still need to be confirmed by further studies.

Nonetheless, the precise biological processes underlying LI remain elusive. Historical data, derived from previous studies, strongly suggest a significant role played by the gut microbiota in the onset of this condition. A study led by MA Azcarate Peril indicates that the genus *Ruminococcus gauvreauii* can offer continuous benefits to LI [52], which is consistent with our MR research conclusions. Another study shows that adding a diet containing probiotics to LI individuals is beneficial for LI patients. Probiotics can significantly improve symptoms such as abdominal pain, bloating, constipation, nausea, and flatulence, and they notably reduce the exhaled H2 levels [53]. Their mechanism of action may be related to the following points: firstly, upon reaching the intestines, probiotics can serve as an essential source of lactase [54], increasing lactose hydrolysis ability and colonic fermentation [55]. Secondly, probiotics counteract abnormal bacterial fermentation in the intestines by secreting antibiotic-like substances [56], adhering to the colonic mucosal surface, enhancing colonic compensatory capacity [57], and can regulate the permeability of the intestinal mucosal barrier [58, 59]. There are also other mechanisms, such as reducing the lactose load [60] and prolonging gastric emptying and the orocecal transit time [61].

Our MR analysis revealed that protective genera, such as *Lachnospiraceae* UCG008, *E. hallii* group, and *Ruminococcus gauvreauii* group, mitigate LI risk likely through modulating gut barrier integrity and inflammatory cell death pathways. These genera may suppress apoptosis and necroptosis by enhancing SCFA production, particularly butyrate, which stabilizes tight junctions (e.g., ZO-1) and promotes anti-inflammatory treg differentiation via HDAC inhibition, counteracting Th17-driven inflammation [14, 62]. Ruminococcus gauvreauii further alleviates osmotic stress by efficiently metabolizing lactose, while reducing mitochondrial ROS accumulation to inhibit FADD-RIPK1-caspase-3 activation [63]. Conversely, the proinflammatory genus *Holdemania* exacerbates LI risk by triggering TLR4/NF-κB-dependent TNF-α release, which promotes RIPK1-mediated necroptosis and disrupts epithelial barrier function through mitochondrial BAX/BAK activation. Critically, chronic Holdemania overabundance may reshape the tumor microenvironment via TGF-β1/SIRT2 signaling, driving fibroblast activation and collagen deposition, a mechanism implicated in premetastatic niche formation [8, 15, 64, 65]. These findings highlight gut microbiota as pivotal regulators of inflammatory crosstalk and cell death pathways in LI, with dual implications for managing intestinal inflammation and cancer risk. Future studies should integrate spatial transcriptomics to resolve region-specific microbiota–epithelial interactions and validate therapeutic strategies targeting SCFA supplementation or RIPK1 inhibition. Limitations include unresolved strain-level functional heterogeneity and potential dietary confounding, necessitating longitudinal validation.

This investigation is underpinned by several pillars of strength. Foremost, when juxtaposed against conventional investigative paradigms, MR analysis is more impervious to confounding element disturbance and reverse causality. Furthermore, our methodological framework sourced genetic constituents from the most exhaustive GWAS meta-survey available on human gut microbiota, consolidating the resilience of our genetic apparatus in this MR scrutiny. To fortify the integrity of our outcomes, we also employed the MR-Egger intercept analysis and the MR-PRESSO technique to inspect potential horizontal pleiotropy that might influence the discerned causal ties.

Nevertheless, our analytical framework is not devoid of potential limitations. At the beginning of our study, we primarily included participants of European descent. This approach helped minimize potential sources of interference but might limit the generalization of our findings to diverse groups. For instance, including solely European-descent participants may lead to selection bias, thereby restricting the extrapolation of our findings. The MR approach employed in our study also has inherent limitations. The instrument variables might suffer from weak correlations and pleiotropy, which could potentially impact the causal inferences of our results. Moreover, there might be other confounding factors unaccounted for in our study design, such as participants' dietary habits and living environment. These factors could possibly interfere with the relationship between gut microbiota and LI. In future research, we could address these limitations by expanding the diversity of the sample population, adopting more precise measurement methods, and incorporating longitudinal studies. The GWAS sequencing results of intestinal flora obtained in this study are based on 16S rRNA sequencing, which can only obtain the classification of flora genera and cannot obtain the information of species. In the future, shotgun metagenomics with a large sample size is conducive to the study from the perspective of species. MR analysis specifically investigates the linear impact of gut microbiota distribution on the susceptibility to LI within diverse populations. Additionally, granular causal interrelationships between gut microbiota variants and LI at the taxonomic species echelon eluded exploration. The ability to detect reverse causality is limited because there are fewer SNPs associated with LI. The intricate biological machinations, underpinning the sway of distinctive gut flora on LI pathogenic evolution, remain clouded in ambiguity. For a more intricate grasp of the ramifications of gut microbiota on LI, nuanced clinical and functional probes are indispensable.

## 5. Conclusions

In conclusion, we thoroughly evaluate the possible cause-and-effect link between gut microbes and LI. This bidirectional MR investigation offers concrete data linking certain gut flora's prevalence with LI. To delve deeper into the influence of probiotics on LI and its underlying biological processes, further studies grounded in robust randomized trials are vital. Moreover, while our reverse MR didn't confirm LI's causal impact on gut flora, the possibility of LI affecting gut organisms remains, warranting additional exploration.

## Figures and Tables

**Table 1:** MR estimates of causal effect of gut microbiota on LI.

Bacterial taxa (outcomes)	MR method	No. SNP	Beta	SE	OR	95%CI	*p*-Value
Genus *Holdemania*	Simple mode	14	0.620	0.063	1.860	0.57–6.06	0.620
	MR-Egger	14	1.008	0.805	2.740	0.57–13.27	0.234
	Weighted median	14	0.616	0.372	1.852	0.89–3.84	0.098
	IVW	14	0.621	0.266	1.860	1.11–3.13	0.019*⁣*^*∗*^
	Weighted mode	14	−0.003	0.025	0.997	0.95–1.05	0.900

Genus *Lachnospiraceae* (UCG008)	Simple mode	11	−0.048	0.502	0.954	0.36–2.55	0.926
	MR-Egger	11	0.715	1.310	2.044	0.16–26.65	0.599
	Weighted median	11	−0.254	0.329	0.776	0.41–1.48	0.440
	IVW	11	−0.538	0.253	0.584	0.36–0.96	0.033*⁣*^*∗*^
	Weighted mode	11	−0.048	0.498	0.954	0.36–2.53	0.926

Genus *Eubacterium* (*hallii* group)	Simple mode	16	−1.111	0.714	0.329	0.08–1.33	0.141
	MR-Egger	16	0.399	0.620	1.490	0.44–5.02	0.530
	Weighted median	16	−0.637	0.414	0.529	0.24–1.19	0.123
	IVW	16	−0.762	0.334	0.467	0.24–0.90	0.023*⁣*^*∗*^
	Weighted mode	16	−0.757	0.599	0.469	0.14–1.52	0.226

Genus *Ruminococcus* (*gauvreauii* group)	Simple mode	12	0.142	0.850	1.1524	0.22–6.10	0.871
	MR-Egger	12	−1.310	1.369	0.270	0.02–3.95	0.361
	Weighted median	12	−0.593	0.472	0.553	0.22–1.40	0.209
	IVW	12	−0.680	0.330	0.507	0.27–0.97	0.039*⁣*^*∗*^
	Weighted mode	12	0.114	0.810	1.121	0.23–5.48	0.890

Abbreviations: CI, confidence interval; IVW, inverse variance weighted; LI, lactose intolerance; ML, maxium likelihood; MR, Mendelian radomization; OR, odds ratio; SNP, single nucleotide polymorphism.

*⁣* ^*∗*^ *p*-value < 0.05.

## Data Availability

The datasets analyzed in this current study can be accessed through the following links: microbiota: https://mibiogen.gcc.rug.nl/ and lactose intolerance: http://r5.finngen.fi/pheno/E4_LACTOSEINT.

## Nomenclature

GWAS: Genome-wide association study
MR: Mendelian randomization
SNP: Single-nucleotide polymorphism
IV: Instrumental variable
IVW: Inverse variance weighted
MR-PRESSO: Mendelian randomization pleiotropy residual sum and outlier
LD: Linkage disequilibrium
LI: Lactose intolerance
OR: Odds ratio
CI: Confidence interval.

## Supporting Information

Supporting Information 1 Figure S1: Leave-one-out plots for the causal effect of LI on gut microbiota. (A) genus *Holdemania*, (B) genus *Lachnospiraceae* (UCG008), (C) genus *Eubacterium* (*hallii* group), and (D) genus *Ruminococcus* (*gauvreauii* group).

Supporting Information 2 Table S1: The selected IVs in the MR study of causal effect of gut microbiota on LI. Table S2: The results of heterogeneity test of gut microbiota IVs. Table S3: The results of directional horizontal pleiotropy test for gut microbiota IVs. Table S4: The selected IVs in the MR study of causal effect of LI on gut microbiota. Table S5: MR estimates of causal effect of LI on gut microbiota. Table S6: The results of heterogeneity test of LI IVs. Table S7: The results of directional horizontal pleiotropy test for LI IVs.
