# Supplementary figures and images for: Gut Microbiota in Lactose Intolerance: A Mendelian Randomization Study on Microbial Mechanisms and Potential Links to Tumor Inflammatory Microenvironments
Source: Mediators Inflamm. 2025 Jun 5;2025:8181816. doi: 10.1155/mi/8181816 (PMC12162160; doi:10.1155/mi/8181816)

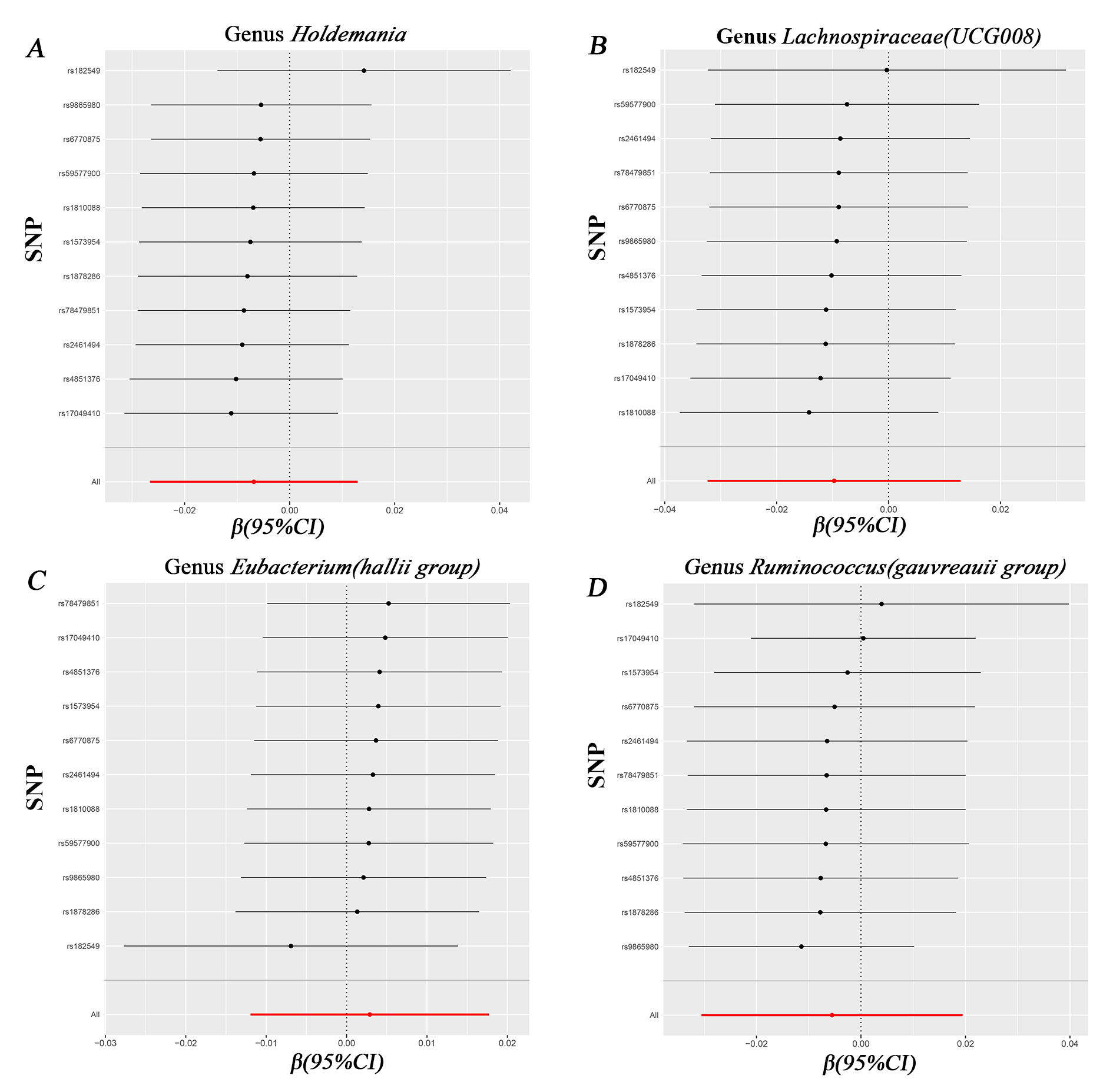

Supplement: Supporting Information 1 — Figure S1: Leave-one-out plots for the causal effect of LI on gut microbiota. (A) genus Holdemania, (B) genus Lachnospiraceae (UCG008), (C) genus Eubacterium (hallii group), and (D) genus Ruminococcus (gauvreauii group). [file 8181816.f1.JPEG]
